# Orbital volume analysis: validation of a semi-automatic software segmentation method

**DOI:** 10.1007/s11548-015-1254-6

**Published:** 2015-07-16

**Authors:** Jesper Jansen, Ruud Schreurs, Leander Dubois, Thomas J. J. Maal, Peter J. J. Gooris, Alfred G. Becking

**Affiliations:** Orbital Unit, Department of Oral and Maxillofacial Surgery, Academic Medical Centre, University of Amsterdam, Meibergdreef 9, 1105 AZ Amsterdam, The Netherlands; Department of Oral and Maxillofacial Surgery, Radboud University Nijmegen Medical Centre, Nijmegen, The Netherlands

**Keywords:** Orbital volume measurement, Pre-operative planning, Orbital fractures, Orbital reconstruction, Validation, Segmentation

## Abstract

**Purpose:**

The purpose of this study was to validate a quick, accurate and reproducible (semi-) automatic software segmentation method to measure orbital volume in the unaffected bony orbit. Precise volume measurement of the orbital cavity is a useful addition to pre-operative planning and intraoperative navigation in orbital reconstruction.

**Methods:**

In 21 CT scans, one unaffected orbit was selected to compare manual segmentation (gold standard) with three segmentation methods using iPlan software (version 3.0.5; Brainlab, Feldkirchen, Germany): automatic (method A), automatic minus bone/air masks (method SA) and automatic minus masks followed by manual adjustments (method SAA). First, validation of the manual segmentation and a newly described method for the anterior boundary was performed. Subsequently the accuracy, reproducibility and time efficiency of the methods were examined. Measurements were performed by two observers.

**Results:**

The intraclass correlation for the interobserver agreement of the anterior boundary was 0.992, and the intraobserver and interobserver agreement for the manual segmentation were 0.997 and 0.994, respectively. Method A had an average volumetric difference of 0.49 cc (SD 0.74) in comparison with the gold standard; this was 0.24 cc (SD 0.27) for method SA and 0.86 cc (SD 0.27) for method SAA. The average time for each method was 38 (SD 5.4), 146 (SD 16.0) and 327 (SD 36.2) seconds per orbit.

**Conclusion:**

The built-in automatic method A is quick, but suboptimal for clinical use. The newly developed method SA appears to be accurate, reproducible, quick and easy to use. Manual adjustments in method SAA are more time-consuming and do not improve volume accuracy. The largest volume discrepancy is located near the inferior orbital fissure.

## Introduction

Reconstruction of the bony orbit is a challenge in post-traumatic orbital wall reconstruction, as well as in the treatment of orbital pathologies such as decompression surgery in Graves’ orbitopathy. The orbit has a complex conical structure [1, 2]. A blow-out fracture is usually the result of trauma to the globe and causes an increase in volume of the bony orbit. An increase of $>$2 cc can lead to significant functional and esthetic sequelae such as diplopia and enophthalmos [3, 4]. Both diplopia and enophthalmos are also seen as a complication after reconstruction of orbital fractures, possibly due to suboptimal anatomical repositioning or reconstruction. An increase of 1 cc in orbital volume is believed to result in 1 mm of enophthalmos on average [1, 5–9]. Other recognized causes of merely late enophthalmos are fat atrophy, fibrosis and loss of periorbital support [10]. Regardless of the approach or choice of materials, restoration of orbital volume to improve function and esthetics should be the main goal [11]. An accurate pre-operative assessment of the orbital content is of importance for achieving an anatomically perfect end result [12]. Precise orbital volume measurement is a useful addition to pre-operative planning for orbital reconstruction, e.g., in traumatology, pathology or decompression surgery.

Computed tomography (CT) is the imaging modality of choice in orbital fractures [13–17]. The quality of CT scanners and reconstruction software has improved significantly over the years. This has enabled the clinician to assess the bony orbit more precise. Despite these improvements, it is still difficult to determine the volume of the bony orbit. The orbital medial wall and orbital floor are very thin structures, and their boundaries are not well defined. This is partly due to the partial volume effect [18, 19]. The position of the anterior boundary is arguable and the posterior boundary is complex with its annulus, inferior and superior orbital fissure and optic foramen.

Several methods to measure orbital volume have been validated over the years [20]. Manual segmentation, outlining the content of the bony orbit slice by slice, is accurate. Unfortunately, it is time-consuming and poorly applicable in clinical practice. In the past, software programs have been developed, with varying results, that are able to segment the orbit (semi-) automatically using CT scans [5, 21–23]. The clinical applicability of a method should depend on how quick, accurate, reproducible and versatile it is [24].

In this study, manual segmentation of the bony orbit, selected to be the gold standard [2, 20], is compared to three different methods using iPlan software (version 3.0.5; Brainlab, Feldkirchen, Germany): the automatic segmentation (method A), a semi-automatic method which combines the automatic method with subtraction of a bone $({\ge }{+}400\hbox { HU})$ and air $({\le }{-}600\hbox { HU})$ density mask (method SA) and a semi-automatic method minus masks combined with manual adjustments (method SAA). The purpose of this study is to validate these (semi-) automatic segmentation methods for measuring orbital volume based on CT scans of unaffected bony orbits and investigate which method is most suitable for clinical and scientific purposes. The manual segmentation and a newly described delineation of the anterior boundary are first validated to make an accurate comparison possible. To our knowledge, the automatic orbital volume segmentation in this software has not yet been validated for orbital volume segmentation. The software possesses functionalities for pre-operative planning and perioperative navigation. The validation of accurate volume analysis serves as a basis for utilizing these functionalities in orbital surgery.

## Materials and Methods

CT data of trauma patients were obtained from the Department of Oral and Maxillofacial Surgery at the Radboud University Nijmegen Medical Centre. From the database of CT scans, a total of 21 orbits, one orbit per scan, was selected. All CT scans were acquired using the standardized trauma protocol (Toshiba Aquillon $\hbox {ONE}^{\mathrm{TM}}$): 0.5 mm slice thickness, 0.5 mm slice increment, 100–120 kV, 80–440 mA, 200–220 FOV, 0.656 Pitch and a $512\,\times \,512$ image matrix. The inclusion criteria were: at least one unaffected bony orbit, no visible orbital pathology, no blood or other body fluids in the ipsilateral sinuses.

### Anatomical boundaries

To calculate a volume in general, a virtually enclosed space is needed. In order to be able to compare the different methods, the orbital boundaries need to be defined first. The anterior boundary is reported to be difficult to define [20]. In this study, interobserver agreement for the anterior boundary was measured using the following method.

**Fig. 1:**
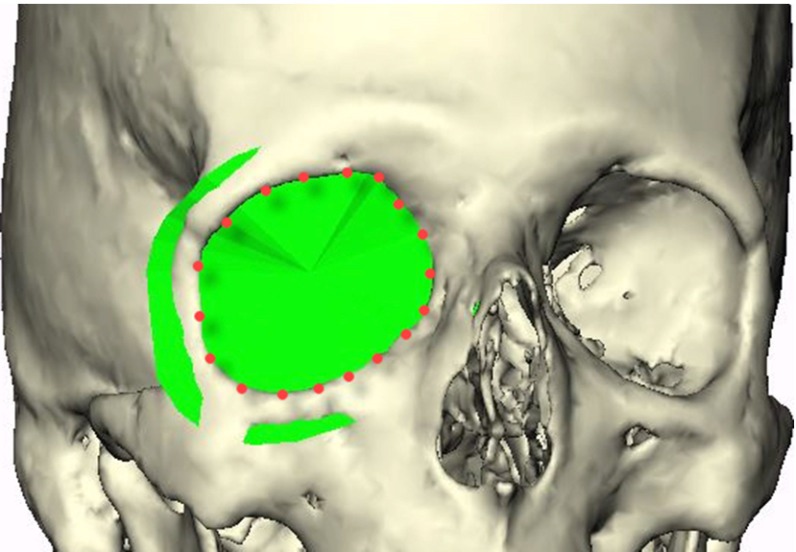
The anterior plane constructed from landmark points positioned along the orbital rim

Two observers placed landmark points along the edge of the orbital rim roughly 0.5–1.0 cm apart from each other using Maxilim software (version 2.3.0; Medicim NV, Mechelen, Belgium) as shown in Fig. 1. This was done for ten scans. The observers started at the supraorbital foramen, continued laterally over the edge of the supraorbital rim, toward the lateral orbital rim and the inferior rim. Medially, the anterior lacrimal crest is followed upward back to the supraorbital foramen. From these indicated landmarks, a surface was reconstructed connecting all landmarks as well as the center of gravity of these landmarks, creating the anterior plane.

The anterior plane, created for each of the 21 orbits, was used as the anterior border in the manual segmentation and each of the (semi-) automatic segmentation methods.

The posterior boundaries of the bony orbit were defined as the initiation of the optic foramen, inferior and superior orbital fissure.

### Gold standard

**Fig. 2:**
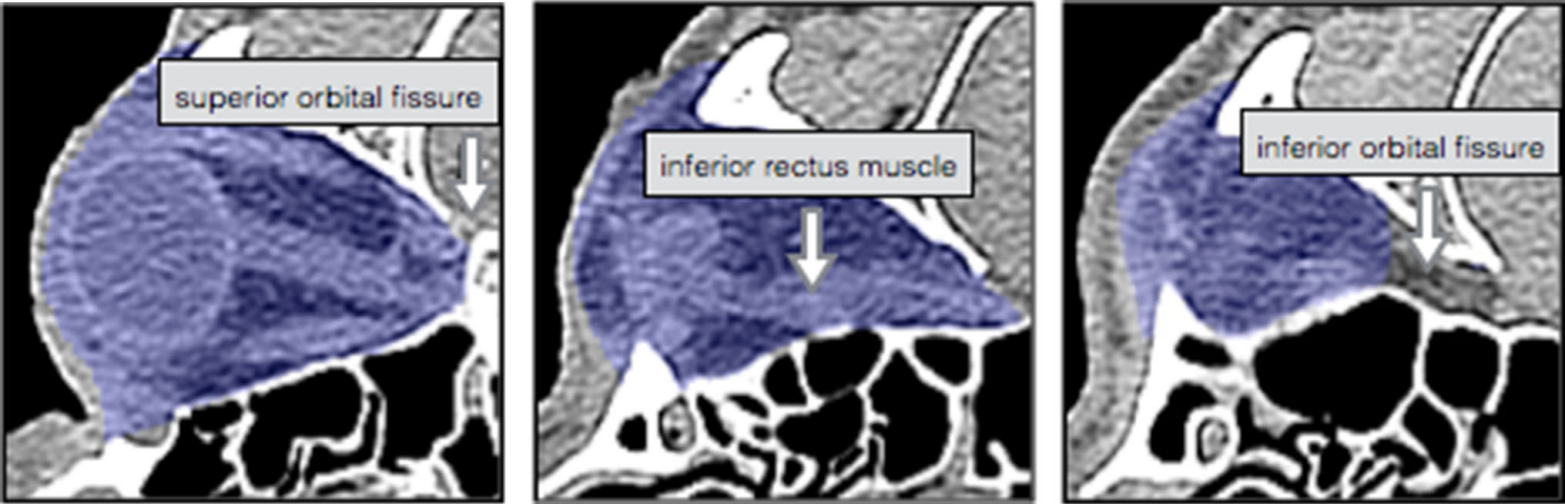
Axial slices of the manual segmentation (gold standard) using a small window ($-200$ and 200HU)

There is no consensus concerning the gold standard for orbital volume measurement. In this study, the manual segmentation of CT scans was used. Initially, the interobserver and intraobserver variability of ten orbits was measured to test the accuracy of this gold standard. Two observers segmented all ten orbits independently; one of the observers performed all segmentations twice. Digital imaging and communication in medicine (DICOM) files of the selected CT scans were imported in Matlab software (version 2012b; The Mathworks Inc., Natick, MA, USA) to perform the manual segmentation. The software used for manual segmentation showed the CT scan in an axial, coronal and sagittal plane, as well as a 3D reconstruction. The window was set to $-200$ to +200 HU to be able to distinguish the different tissues. Moving caudally, the orbital volume was segmented by tracing the orbital boundaries manually in each individual axial slice. The initial segmentation was performed in the axial slices and followed by adjustments in the coronal and sagittal direction, if necessary. The extraocular rectus muscles were traced apically to determine the posterior boundary of the apex (Fig. 2). The segmented volumes were imported in Maxilim, and a reconstruction of the segmentation was generated to obtain a virtual model of the orbital content. Excess anterior volume of the reconstructed model was removed according to the aforementioned anterior plane, and a volumetric measurement of this cleaned model was generated within the software.

### (Semi-) automatic segmentation methods

**Fig. 3:**
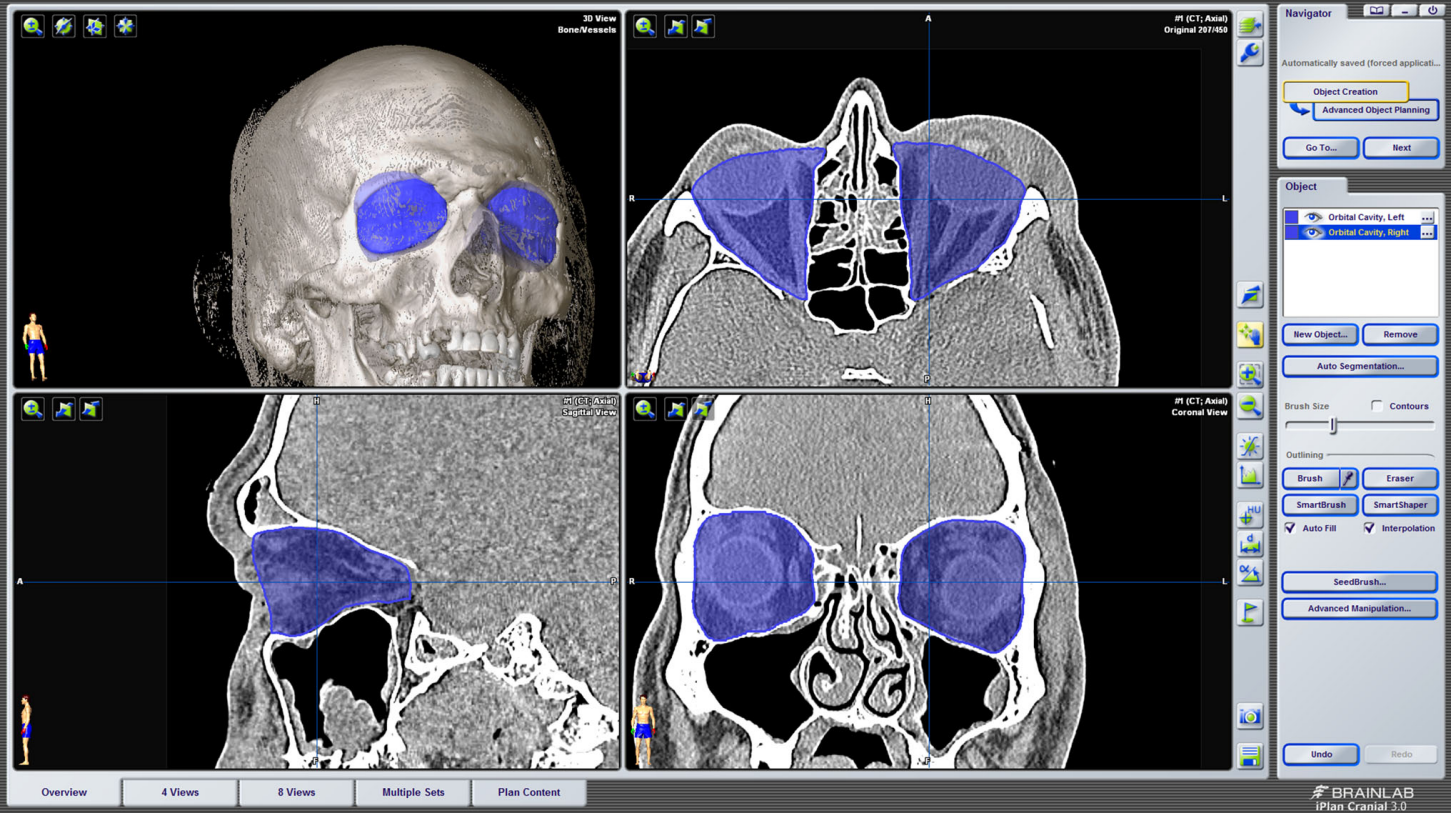
Screenshot of the automatic segmentation in iPlan 3.0.5

Three different segmentation methods were used to measure orbital volume using iPlan 3.0.5 after importing the DICOM files (Fig. 3):

1. Method A (automatic): automatic segmentation of the orbital cavity by means of the built-in functionality in the software. The automatic segmentation is established by atlas-based segmentation. This method uses prior information of training images to recognize the shape and gray levels of determined parts of the body to perform auto-segmentation [25, 26].
2. Method SA (semi-automatic): the automatic segmentation with subtraction of bone and air density masks. A bone mask (+400 HU or more) and air mask ($-600$ HU or less) were created and subtracted from the segmentation that was obtained by the automatic method.
3. Method SAA (semi-automatic with manual adjustments): the automatic method with subtraction of bone and air mask, followed by manual adjustment of large errors using the smart shaper tool and eraser (both built-in functionalities in the software). First, the position of the scan was altered so that the skull was in a true horizontal position. The window was set between $-200$ and +200 HU. The axial slices were quickly scanned for significant irregularities and mistakes in added voxels outside the bony orbit. Then the sagittal plane was used to delineate the inferior orbital fissure by following the inferior rectus muscle and to define the apical limit. Finally, the axial slices were scanned to make final adjustments.

Final volume calculations were performed in Maxilim. In Maxilim, the anterior planes previously used for the manual reconstructions (gold standard) were used to remove content outside the bony orbit of the (semi-) automatic methods in order to measure the volume and compare it to the gold standard.

### Statistical analysis

The results in this article were analyzed in IBM SPSS Statistics (version 22.0; IBM Corp., Armonk, NY, USA). The volumes for the anterior boundary and manual segmentation were computed, and descriptive statistics, mean difference and standard deviation (SD), were calculated. The intraclass correlation coefficient (ICC) and 95 % confidence interval (CI) were measured to test the interobserver and intraobserver agreement for the volumetric measurements for both the anterior boundary as well as the manual segmentation (gold standard).

For the (semi-) automatic segmentation methods, the correlation between the gold standard and the computed volumes of the three separate methods was analyzed using the mean difference and SD. For the SAA method, both interobserver and intraobserver agreement were measured using ICC and the 95 % CI of the bony orbital volumes. The average time in seconds and SD was also calculated for all three methods. Finally, Dice coefficients and distance maps were computed to compare the (semi-) automatic methods to the gold standard. For the distance maps, the mean difference on the border of the segmentation was compared in millimeters (mm). Both mean distance measure and the 95th percentile of the absolute distance measure were measured. Differences in the data were tested using a paired student’s *t* test.

## Results

### Validation of the anterior boundary

Two observers indicated the landmark points on ten CT scans. The ICC was 0.992 (95 % CI 0.956–0.998) when comparing the orbital volumes after the cutoff by the anterior planes reconstructed by the different observers for all datasets. The mean difference for the resulting volumes was 0.17 cc (SD 0.24).

### Validation of the gold standard

Two observers performed the manual segmentation for ten scans. The mean volume of all calculations was 29.9 cc (SD 2.26). The intraobserver ICC was 0.997 (95 % CI 0.987–0.999) with a mean difference of 0.09 cc (SD 0.18). The interobserver ICC was 0.994 (95 % CI 0.976–0.998) with a mean difference of 0.03 cc (SD 0.27).

### Gold standard versus three (semi-) automatic segmentation methods

All 21 orbits were segmented to compare the gold standard to the three different methods (Table 1). Method A had a mean difference of 0.49 cc (SD 0.74) in comparison with the gold standard. The average time was 38 s (SD 5.4) per orbit. Method SA had a mean difference of 0.24 cc (SD 0.27) with the gold standard segmented orbits. The average time was 146 s (SD 16.0) per orbit. Method SAA gave a mean difference of 0.86 cc (SD 0.27) to the gold standard segmentation. The average time was 327 s (SD 36.2) per orbit. The ICC for the intraobserver variability was 0.998 (95 % CI 0.991–0.999) and the interobserver variability 0.990 (95 % CI 0.890–0.998) for method SAA.

**Table 1:** Results of the three (semi-) automatic methods compared to the gold standard

	Average difference (cc)	SD (cc)	Intraobserver (ICC)	Interobserver (ICC)	Average time (s)	SD (s)
Method A	0.49	0.74	–	–	38	5.4
Method SA	0.24	0.27	–	–	146	16.0
Method SAA	0.86	0.27	0.998	0.990	327	36.2

**Fig. 4:**
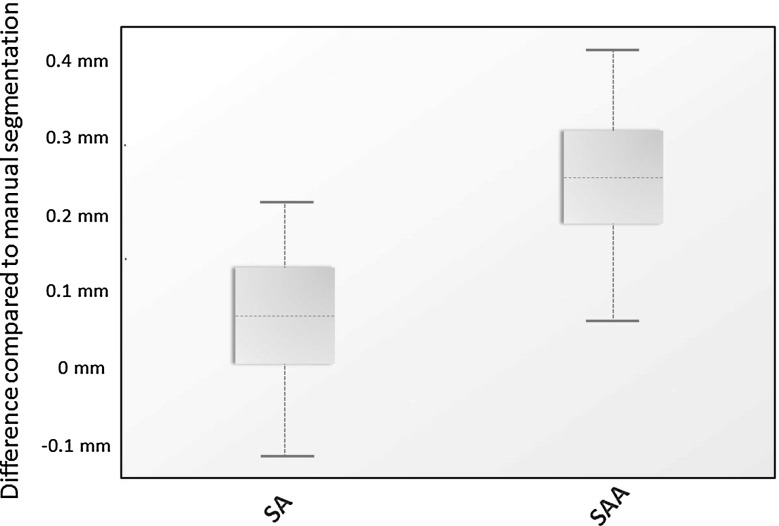
Mean distance measure between method SA versus gold standard and method SAA versus gold standard $(p<0.001)$

**Fig. 5:**
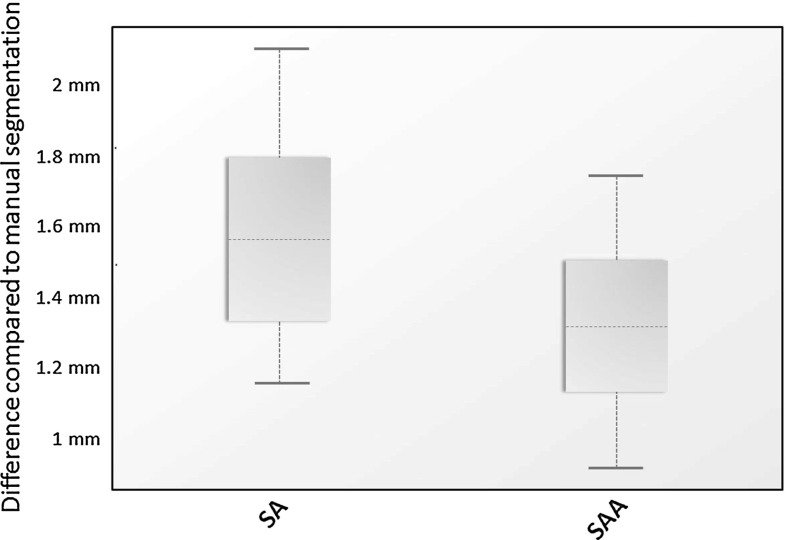
95th percentile of the absolute distance measure between method SA versus gold standard and method SAA versus gold standard ($p=0.001$)

Concerning the distance maps, the mean distance measure for method SA was 0.07 mm (SD 0.09) and for method SAA 0.24 mm (SD 0.10) compared to the gold standard. Paired Student’s *t* test showed a significant difference $(p<0.001)$ (Fig. 4). The 95th percentile of the absolute distance measure of method SA was 1.58 mm (SD 0.30) and 1.33 mm (SD 0.23) for method SAA. The SAA method showed a significantly larger difference to the gold standard segmentation than the SA method ($p=0.001$; Fig. 5). The mean Dice coefficients of method A, method SA and method SAA each compared to the gold standard were 0.961 (SD 0.011), 0.973 (SD 0.003) and 0.979 (SD 0.003), respectively.

## Discussion

The purpose of this study was to validate a quick, accurate and reproducible (semi-) automatic segmentation method to measure orbital volume of unaffected bony orbits. The orbit is a complex anatomical structure, which makes it challenging to measure its volume accurately. Not only does it have thin walls, it also lacks an anterior border and has several posterior anatomical gaps. The anatomy becomes even more complex if an orbital wall is fractured. For this reason, it is important to optimize pre-operative diagnostics to improve outcome after orbital reconstruction. In our opinion, accurate measurement of the orbital volume is the first step in pre-operative planning.

### Anterior boundary

The anterior part of the orbit has the widest diameter and is therefore responsible for the biggest deviation in volume measurement, even with small differences in diameter estimations. In this study, a new method was validated for the anterior boundary of the bony orbit. A surface was reconstructed connecting all landmarks as well as the center of gravity of these landmarks, creating the anterior plane. This is congruent with the description of the anterior border shape of Osaki et al. [20]. Our study is the first study demonstrating the accuracy and reproducibility of this method. The anterior plane was used to separate excess volume outside the bony orbit in all segmentations in order to be able to compare them. This eliminated any doubts about volume differences at the anterior boundary.

### Gold standard

In the literature, two methods are suggested to be the gold standard for measuring orbital volume: slice-by-slice manual segmentation of CT scans and the use of different kinds of filling materials (beads, silicone, water) for the enucleated orbits of cadavers [20]. Both methods have their advantages and disadvantages. The advantage of the manual segmentation is that only a CT scan is required to measure the volume. The disadvantage is that it still is an observer-dependent process, and therefore, it is subject to discrepancies in assessment between observers. The filling method has the advantage that a real volume is measured. The disadvantage is that it is difficult to contour the anterior border of the orbit, which means that it is practically impossible to measure the exact orbital volume. Apart from this, the method can only be used in anatomical specimen and is useless in a clinical situation. In this study, the manual segmentation method was used as the gold standard. The reproducibility of the method investigated was validated and demonstrated sufficient high correlation for both interobserver and intraobserver measurements. Trauma scans were used on purpose to mimic the clinical pre-operative setting. This means that the patient was not always scanned in a well-aligned position. The agreement may have been even higher when scan data of properly aligned patients had been used.

### (Semi-) automatic methods

As mentioned in the introduction, 2 cc increase of orbital volume leads to 2 mm of enophthalmos on average, which is considered to be clinically significant. Accuracy and reproducibility should be well within those limits to prevent measuring errors from contributing to poor surgical outcome due to planning. In the past two decades, several (semi-) automatic software methods have been tested with varying results [5, 21–23]. This is partly due to the differences in choice of gold standard, approach and study design. It is difficult to compare results from these studies. A study by Deveci et al. [23] was one of the first to compare a 3-dimensional reconstructive software program to a gold standard for direct measurements. A filling method (alleged gold standard in that study) was compared to a 3D software program. They reported no significant volume discrepancy between the two methods, but the accuracy was not acceptable compared to findings in the recent literature. The mean volume difference was 0.93 cc (SD 1.08) and therefore insufficient for clinical use considering how this relates to enophthalmos. Regensburg et al. [21] compared direct measurement with a CT-based method in Mimics version 9.11 to measure bony orbital volume and orbital fat/muscle volume. This was performed on a single phantom and showed a difference of $-0.7$ and +0.7 % in fat and $-1.5$ and $-2.2$ % in muscle volume compared to the known volume. No statements were made on the total bony orbital volume of the phantom. Intraobserver variability was ${<}5$ % for the calculations of fat volume, muscle volume and bony orbital volume. This represents approximately 1.5 cc of total orbital volume, which can be considered a substantial measuring error. Strong et al. published very small intraoperator and interoperator errors when using Maxillo software [5]. However, comparison with a gold standard is lacking, so it is impossible to know if the real volume was measured.

In Method A, the built-in automatic segmentation was not accurate enough, probably due to the many morphological challenges hindering accurate segmentation. Method A was easy, fast and reproducible. However, it often overestimated the volume as it frequently included parts of the surrounding bone, air (frontal/ethmoidal sinus) and inferior orbital fissure in the segmentation. This resulted in a mean difference of 0.49 cc (SD 0.74) compared to the gold standard. Therefore, this auto-segmentation is not advisable in a clinical setting.

In the newly developed method SA, bone and air masks were created. The method was designed to solve the problem of overestimation due to inclusion of bone and air in the segmentation. This resulted in higher accuracy, while the time needed to perform the segmentation increased only slightly. Mean difference compared to the gold standard was 0.24 cc (SD 0.27) and average time 146 s (SD 16.0). The SA method was still perfectly reproducible, because the creation of the mask is not observer dependent.

**Fig. 6:**
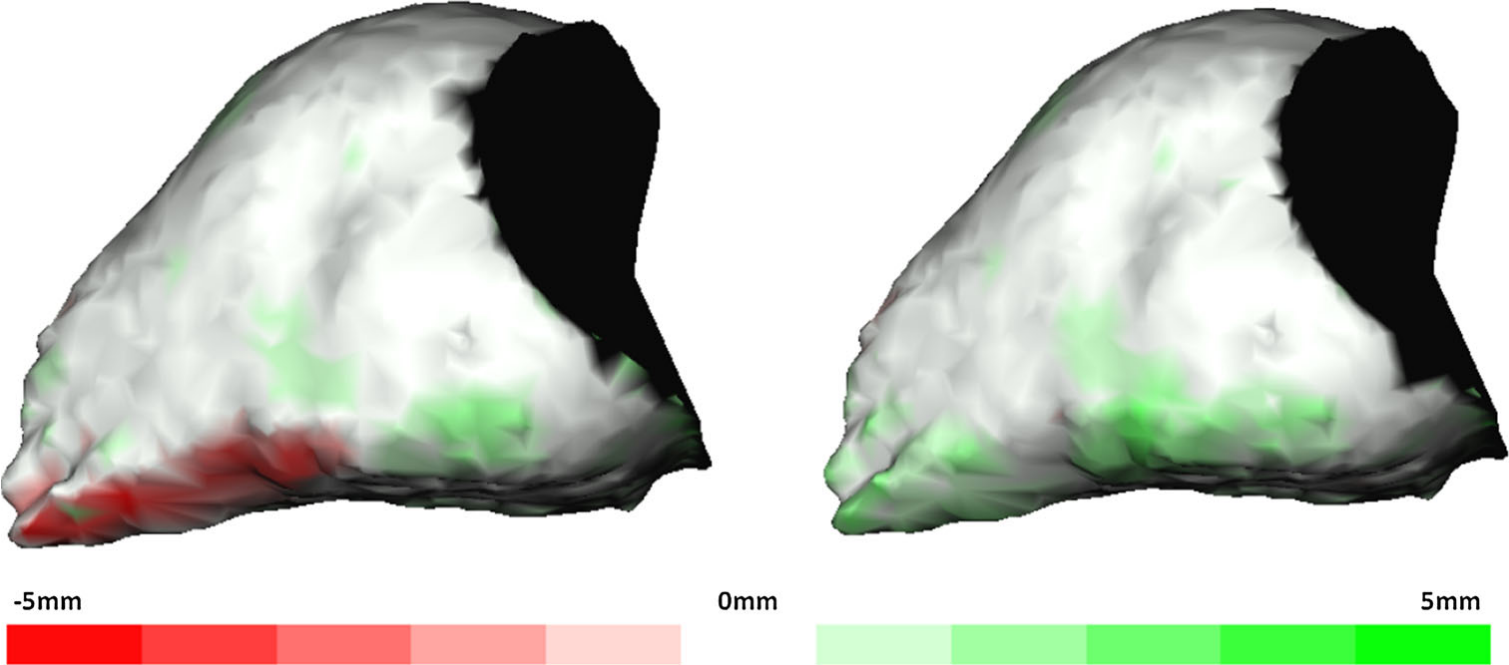
Distance maps of method SA (*left*) and method SAA (*right*) projected on the gold standard segmentation; *red* illustrates excess and *green* deficiency in volume of the (semi-) automatic methods compared to gold standard

Differences in volume between the (semi-) automatic methods and the gold standard are greatly influenced by differences in defining the border of the inferior orbital fissure (Fig. 6). To overcome this repeating error within the automatic method, a manual adjustment was introduced in method SAA. It was thought that this would correct overestimation and prevent large errors. Unfortunately, it consistently produced an underestimation and had poorer accuracy with a mean difference of 0.86 cc (SD 0.27) compared to the gold standard. The reproducibility of this method was acceptable, but worse than the other two (semi-) automatic methods. Furthermore, method SAA is more time-consuming with average time of 327 s (SD 36.2). The semi-automatic method without manual adjustments proved to be accurate with an average difference of 0.24 cc (SD 0.27) compared to the gold standard.

The distance map results of the mean distance measure between method SA versus gold standard and method SAA versus gold standard illustrated that the dataset of method SA had a better general fit compared to the gold standard. The 95th percentile of the absolute distance measure of both datasets showed that method SAA had less outliers than method SA compared to the gold standard. This concludes that method SA has the best fit in comparison with the gold standard; however, it is more susceptible to large differences in specific areas than method SAA. Inspection of the distance maps showed that most outliers were situated near the inferior orbital fissure.

The results of method SAA are surprising, as one would expect that slight manual adjustments would improve method SA. The results of the distance maps and Fig. 6 for method SA show an accurate resemblance to the gold standard in most regions. The only region that is different is the region around the inferior orbital fissure, where a volume increment is seen in the SA model. The distance map of method SAA (Fig. 6) corresponds to that of method SA, except for the inferior orbital fissure, which now shows a volume decrement compared to the gold standard. An underestimation of the total volume was seen in method SAA for both observers, probably due to overcorrection of the orbital contour by the use of the built-in smart shaper tool and difficulty to find the border of the orbital volume and inferior orbital fissure. The smart shaper tool intelligently facilitates recontouring of the segmented volume in 3D by working on a 2D slice. This saves time, as not every slice has to be altered one at the time. However, this probably caused the overcorrection as alterations were made to other slices without accurate control [27].

The Dice coefficients of all three methods compared to the gold standard are excellent with method SAA being slightly better than method SA. The outliers in method SA described earlier might explain this. Method SAA showed a slightly better overlap between the segmentation by means of the Dice coefficient. Nevertheless, method SA proves to be superior considering the volume accuracy, reproducibility, and time efficiency compared with a high Dice coefficient.

## Conclusion

In conclusion, in this study a manual segmentation, anterior boundary and three methods using iPlan 3.0.5 were validated for the unaffected bony orbit. The results showed that method SA (automatic segmentation with subtraction of bone and air density masks) can be highly recommended based on the results of the study. This method proved to be accurate, reproducible, quick and easy to use. The automatic segmentation option should only be used in combination with educated inspection afterward. This is mainly because of errors due to automatically adding volume of bony and pneumatized areas, as well as added volume of the inferior orbital fissure.

The accuracy of orbital reconstructive surgery will benefit from improvements in diagnostics and planning using 3D software. Apart from experience and surgical skills, outcomes of orbital reconstruction depend on careful and precise measurements and planning in the pre-operative assessment, intraoperative navigation and intraoperative radiography. Method SA could provide better pre-operative assessment and might therefore result in fewer complications and less need for secondary reconstructions.

The researchers are aware that many additional aspects, such as post-traumatic and iatrogenic fat atrophy, fibrosis and adhesions, may affect the outcome of orbital surgery. It is believed to be possible to exactly restore the volume of the bony orbit, but changes in the orbital content may compromise the final result. Nevertheless, the extent of these factors is difficult to analyze without adequate orbital volume measurements. In pre-operative assessment, correct and accurate orbital volume calculation should be part of diagnosing orbital pathology and (virtual 3D) planning of orbital reconstructions. A next challenge is volume segmentation in patients with an orbital fracture. Future steps may be segmentation and manipulation, e.g., implementation of mirroring of the unaffected contralateral orbit. Further studies are being performed to validate the benefits of these new methods.
